# From armed-conflict to care: clinical outcomes, diagnostic challenges, and psychosocial burden in children evaluated for suspected cancer displaced from Gaza and treated at a regional center of excellence

**DOI:** 10.3389/fonc.2026.1934075

**Published:** 2026-10-09

**Authors:** Rawad Rihani, Shrouq Amer, Maha Barbar, Omar Shamieh, Arwa Kiswani, Richard Sullivan, Carlos Rodriguez-Galindo, Asem Mansour

**Affiliations:** 1 Department of Pediatrics, King Hussein Cancer Center (KHCC), Amman, Jordan; 2 Department of Gastroenterology and Nutrition, King Hussein Cancer Center (KHCC), Amman, Jordan; 3 Department of Palliative Care, King Hussein Cancer Center (KHCC), Amman, Jordan; 4 Institute of Cancer Policy, King’s College London, London, United Kingdom; 5 St Jude Children’s Research Hospital, Memphis, TN, United States; 6 Chief Executive Office, King Hussein Cancer Center (KHCC), Amman, Jordan

**Keywords:** armed-conflict, diagnostic concordance, displacement, Gaza, pediatric oncology

## Abstract

**Background:**

Conflict-related displacement profoundly disrupts pediatric cancer care, yet clinical outcomes data for evacuated children treated at regional oncology centers remain scarce. We describe the clinical characteristics, diagnostic challenges, nutritional and psychosocial burden, and outcomes of children evacuated from Gaza and treated at King Hussein Cancer Center (KHCC), Jordan.

**Methods:**

We conducted a retrospective cohort study of patients aged ≤21 years evacuated from Gaza to KHCC between November 2023 and March 2026. Demographic and clinical characteristics, cancer type and stage, diagnostic concordance, nutritional and psychosocial status, and survival outcomes were assessed. Overall survival (OS) and event-free survival (EFS) were estimated using Kaplan–Meier methods.

**Results:**

A total of 169 patients were included; median age was 6.7 years (range, 0.01–21.3), and 62% were male. Median time from symptom onset to presentation was 3 months (range, 1–62). Among staged solid tumors, lymphomas, and brain tumors, 41.6% had distant disease at presentation. All patients with leukemia presented with systemic disease at diagnosis, and all patients with retinoblastoma presented with moderate-to-advanced intraocular disease (International Intraocular Retinoblastoma Classification Groups C–E), with 83% classified as high-risk (Groups D/E). Diagnostic discordance occurred in 19.5% of cases. Nutritional and psychosocial burden was substantial: 26.3% were underweight (44/167 assessed), 50.3% had vitamin D deficiency (75/149 assessed), and 41.4% reported clinically significant psychological distress. During a median follow-up of 8.0 months, 24 patients died (14.2%), including 2.4% who died within 30 days of presentation. One- and 2-year OS were 81.3% (95% CI, 72.1–87.7%) and 75.7% (95% CI, 64.6–83.7%), respectively; corresponding EFS estimates were 76.5% (95% CI, 67.0–83.6%) and 70.6% (95% CI, 59.4–79.3%). PICU-admitted patients had markedly lower 1-year OS (52.7% vs 86.8%; HR, 5.56; log-rank P<0.0001) and EFS (48.9% vs 81.8%; HR, 4.27; log-rank P<0.0001).

**Conclusions:**

Children evacuated from Gaza presented with advanced disease, diagnostic discordance, malnutrition, and substantial psychosocial burden, reflecting the clinical consequences of conflict-related disruption of healthcare. Despite these challenges, multidisciplinary care at a regional center of excellence achieves meaningful survival outcomes. These findings underscore the need for protected humanitarian evacuation pathways, integrated supportive care, and sustainable international funding for pediatric oncology services in conflict settings.

## Introduction

Childhood cancer is one of the most common causes of death from disease worldwide, with approximately 400,000 new cases diagnosed annually (1, 2). Nearly 80–90% of children with cancer live in LMICs, where inequities in access to timely diagnosis and treatment contribute to persistently poor survival outcomes (3), driven by delayed diagnosis, restricted access to treatment, and health system limitations (4, 5). These disparities are further exacerbated in conflict and forced displacement settings, where disruption of healthcare infrastructure, restricted mobility, and resource scarcity are associated with significant impact on cancer care delivery (6). Between 2020 and 2050, an estimated 13.7 million children worldwide will be diagnosed with cancer, most in resource-scarce settings, and without urgent systems strengthening and conflict reduction, 11.1 million are projected to die (7).

Children affected by armed conflict represent a particularly vulnerable population (8). Gaza exemplifies this vulnerability at an extreme scale: the escalation of conflict from October 2023 has severely degraded an already fragile healthcare system, limiting cancer services and disrupting routine immunization programs (9). Children with suspected or confirmed cancer are frequently evacuated to neighboring countries (9, 10), resulting in substantial diagnostic and treatment delays and advanced disease at first presentation (11, 12). Despite the growing number of conflict-displaced children with cancer, data on their clinical characteristics, diagnostic accuracy, and post-evacuation outcomes remain limited.

The King Hussein Cancer Center (KHCC) has served as a regional referral center for displaced children with cancer. It has provided care through coordinated humanitarian efforts with international partners, such as St. Jude Children’s Research Hospital (SJCRH) (13).

In addition to clinical challenges, these children carry a substantial psychosocial burden compounded by displacement, and conflict-related trauma (14, 15), while accompanying caregivers frequently present with unaddressed medical and psychological needs of their own. This study characterizes the demographic and clinical features of pediatric patients from Gaza referred to KHCC, with a focus on diagnostic concordance, disease stage at presentation, early outcomes, high-risk subgroup requiring intensive care and psychosocial and nutritional burden.

## Methods

### Study design and population

This retrospective cohort study was approved by the KHCC Institutional Review Board with a waiver of informed consent (IRB #24KHCC213); all data were de-identified. The cohort comprised all pediatric patients (aged ≤21 years) from Gaza evaluated for suspected malignancy at KHCC between November 2023 and March 2026 following medical evacuation to Jordan; the cohort does not include all evacuated pediatric patients broadly. **Supplementary Figure 1** shows the patient flow from referral to final diagnostic outcome and the denominators used for each subsequent analysis. Non-malignant conditions were included in descriptive analyses but excluded from stage-at-presentation analyses; leukemia (systemic by definition) and retinoblastoma (staged by the International Intraocular Retinoblastoma Classification (IIRC) rather than Surveillance, Epidemiology, and End Results (SEER)) were also excluded from the pooled SEER staging denominator and are reported separately.

Data were extracted from electronic medical records and included demographics, clinical timelines (symptom onset, diagnosis, and referral), disease characteristics, treatment history, and outcomes. Cancer diagnoses were classified per the International Classification of Childhood Cancer, Third Edition (ICCC-3) (16), and staging used the SEER Program system (17). Diagnostic concordance was assessed between the referring and final KHCC diagnoses. Referring diagnoses from Gaza represented provisional working diagnoses based on the clinical, imaging, and — where available — pathology information accompanying the patient; most patients arrived with documentation (clinical/pathology reports) but without physical tissue. Where original pathology material (slides/blocks) accompanied the patient, it was reviewed by KHCC pathology; in its absence, the referring diagnosis was assessed against the diagnosis subsequently established at KHCC. The KHCC diagnosis — based on complete pathology review, immunophenotyping/molecular work-up, and imaging performed at KHCC — was adopted as the reference standard, reflecting the substantially greater diagnostic information available at the receiving center rather than an assumption that it was infallible. Discordant cases were adjudicated through multidisciplinary tumor board review and categorized as reclassification of cancer type, reclassification to a non-malignant condition, or non-confirmation of suspected relapse. Nutritional status was assessed via weight-for-age percentiles and serum markers (albumin, pre-albumin, vitamin D, and vitamin B12); testing rates varied by marker and are reported per-test rather than assuming universal ascertainment. Psychological distress was assessed using the Distress Thermometer scale (DTS) (18) by a trained psychologist or social worker following an interview with the child and caregiver. For younger children who were unable to provide a reliable self-report, assessment incorporated caregiver input and the clinical interview. A score ≥6 was used to identify clinically significant distress, consistent with the cited literature (19). Accompanying caregiver characteristics, administrative evacuation barriers, and funding source data were also collected. Patients with non-malignant conditions were included in descriptive analyses but excluded from staging and survival analyses. Funding source was extracted from institutional billing and financial records and categorized according to the organization supporting the patient’s care.

### Statistical analysis

All statistical analyses were performed using R version 4.3.1 (R Foundation for Statistical Computing, Vienna, Austria), with the survival (version 3.5) and survminer (version 0.4.9) packages for Kaplan–Meier estimation and Cox proportional hazards modeling. For continuous variables, we calculated the median and the interquartile range (IQR). For categorical variables, we calculated the frequency and percentage. Categorical variables were compared between groups using Fisher’s exact test; continuous variables using the Mann–Whitney U test. Missing data were handled by exclusion from the denominator for the affected variable.

Overall survival (OS) was defined as time from KHCC presentation to death from any cause. Event-free survival (EFS) was defined as time from KHCC presentation to the first subsequent occurrence of relapse, disease progression, or death from any cause. For patients presenting with relapse or progressive disease, the presenting disease status was considered the baseline state rather than an EFS event. A subsequent event was recorded only if a new event occurred after KHCC presentation, such as death, a new relapse following achievement of remission, or subsequent disease progression. Patients who remained free of a subsequent event were censored at the date of last documented follow-up. Because patients entered the cohort at different points in their disease trajectories, including newly diagnosed, previously treated, and relapsed/progressive disease, survival time was anchored to KHCC presentation rather than original cancer diagnosis. Survival curves were estimated by the Kaplan–Meier method and compared with the log-rank test. A univariable Cox proportional hazards model was used to estimate the hazard ratio for pediatric intensive care unit (PICU) versus non-PICU admission; this model is unadjusted for disease stage, tumor type, and other clinical confounders. Survival is reported at 1 year and 2 years, consistent with the observed follow-up period (median 8.0 months; maximum approximately 28 months; data lock March 31, 2026). Early mortality was defined as death within 30 days of presentation.

## Results

### Patient characteristics

A total of 169 pediatric patients from Gaza were evaluated at KHCC between November 2023 and March 2026. The cohort was predominantly male (62%, n=105), with a median age of 6.7 years (range, 0.01–21.3); 40% were under 5 years, 27% aged 5–10 years, and 33% aged ≥10 years. Median follow-up was 8.0 months (IQR 5.2–22.0) (**Table 1**).

**Table 1:** Baseline characteristics of pediatric patients from Gaza evaluated at KHCC (N = 169).

Characteristic	n (%)
Sex	
Male	105 (62%)
Female	64 (38%)
Age group	
<5 years	68 (40%)
5–10 years	45 (27%)
≥10 years	56 (33%)
Referral indication	
Suspected new cancer diagnosis	148 (88%)
Suspected relapse	21 (12%)
Prior treatment in Gaza	
Yes	85 (50%)
No	84 (50%)
Referral diagnosis¹	
Solid tumors	56 (33%)
Acute leukemias	40 (24%)
Lymphomas	30 (18%)
CNS tumors	24 (14%)
Retinoblastoma	9 (5%)
Histiocytic disorders	7 (4%)
Hematologic disorders (non-malignant)	3 (2%)
Disposition at arrival	
Outpatient	103 (61%)
Admission	66 (39%)
PICU	23 (35%)^2^
Floor	43 (65%)^2^
Disease status after evaluation at KHCC	
Remission	60 (36%)
Continue treatment	42 (70%)^2^
Observation/follow-up	18 (30%)^2^
Relapse/progressive disease	96 (57%)
Non-cancer diagnosis	13 (8%)
Funding source	
King Hussein Cancer Foundation (KHCF)	100 (59%)
St. Jude Children’s Research Hospital (SJCRH)	69 (41%)

¹Referral diagnoses reflect initial classification prior to KHCC evaluation. Disease status is assessed at time of arrival to KHCC (pre-treatment), distinct from **Table 2**’s final diagnosis category assigned after complete KHCC work-up; the non-cancer total here (n=13) includes 9 patients also captured in **Table 2**’s “Other/non-cancer” category (n=11 total; the remaining 2 arrived with disease status recorded as relapse/progression but were subsequently reclassified as non-malignant at KHCC), 2 patients with non-malignant hematologic disorders (aplastic anemia, transfusion-dependent thalassemia), and 2 patients in whom suspected relapse was ruled out at arrival and who are retained under their original cancer diagnosis category in **Table 2**, ^2^Percentage of subgroup. KHCC, King Hussein Cancer Center; PICU, pediatric intensive care unit.

At referral, 88% (n=148) were suspected new cancer diagnosis and 12% (n=21) suspected relapse. Half (50%, n=85) had received prior cancer-directed therapy (chemotherapy, radiotherapy, and/or surgery) in Gaza, though treatment details were frequently incomplete. At presentation, 61% (n=103) were managed as outpatients and 39% (n=66) required admission, including 14% (n=23) directly to the PICU. (**Table 1**) Median time from symptom onset to KHCC presentation was 3 months (range 1–62). This interval reflects the total elapsed time from symptom onset to KHCC assessment and encompasses any care received in Gaza prior to evacuation.

### Disease characteristics and diagnostic concordance

The most common referral diagnoses were solid tumors (33%, n=56), acute leukemia (24%, n=40), lymphomas (18%, n=30), and CNS tumors (14%, n=24) (**Table 1**). Following KHCC evaluation, 57% (n=96) had active or progressive disease, 36% (n=60) were in remission, and 8% (n=13) were diagnosed with non-malignant conditions. (**Figure 1A**).

**Figure 1:**
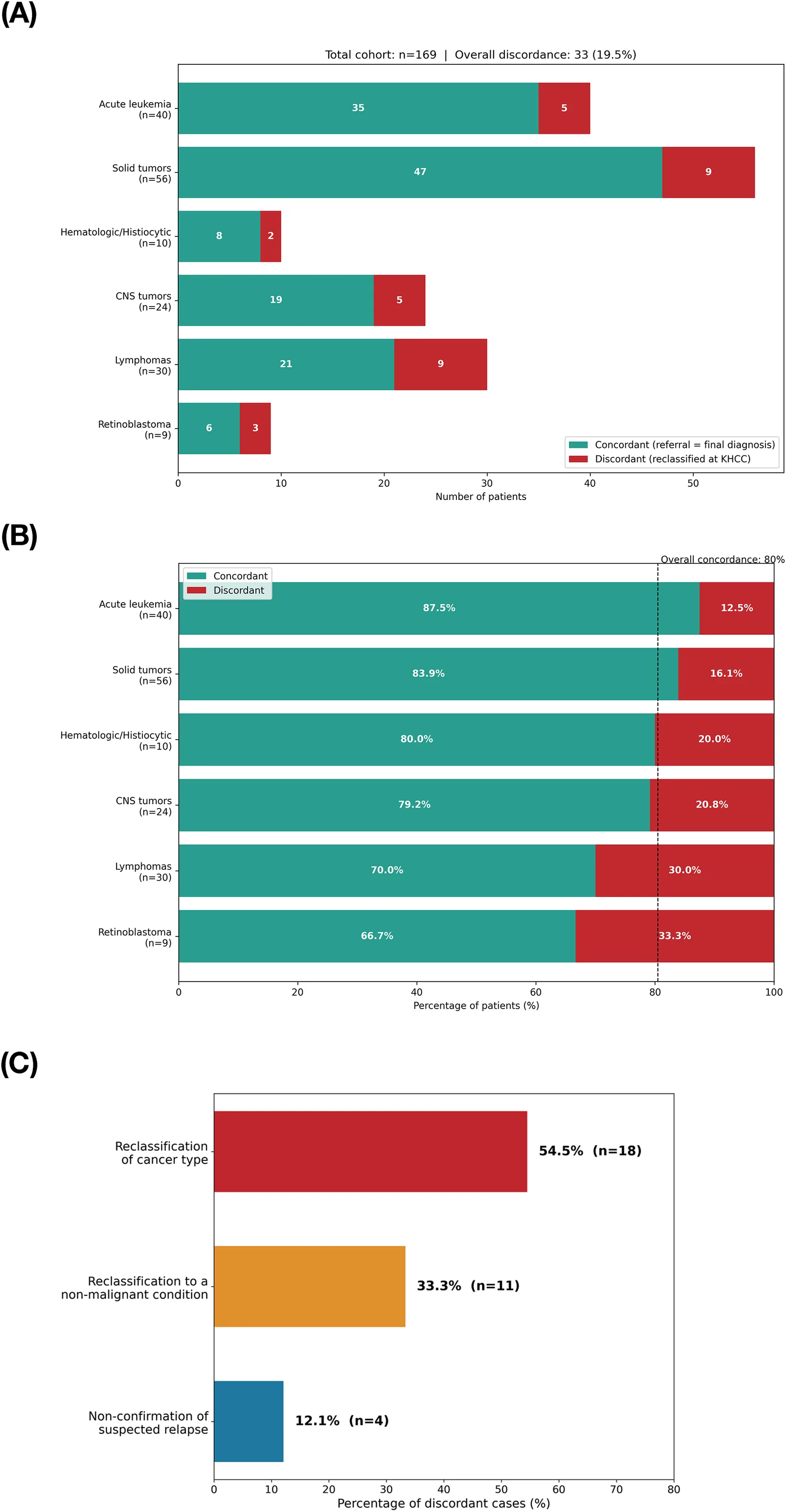
**(A)** Diagnostic concordance between Gaza referral diagnosis and final diagnosis at KHCC, by tumor category. Stacked bar chart showing absolute patient counts of concordant and discordant diagnoses per disease category (n=169). Numbers within bars represent patient counts. Categories are sorted by concordance rate in descending order. Discordant cases include reclassification of cancer type; reclassification to a non-malignant condition; non-confirmation of suspected relapse. KHCC, King Hussein Cancer Center. **(B)** Diagnostic concordance rates between Gaza referral diagnosis and final KHCC diagnosis, by tumor category. Stacked bar chart showing the proportion of concordant and discordant diagnoses per disease category. The dashed vertical line represents the overall cohort concordance rate (80%). Categories are sorted by concordance rate in ascending order. KHCC, King Hussein Cancer Center. **(C)** Distribution of discordance types among 33 discordant cases (19.5% of total cohort). Horizontal bar chart showing the proportion of each discordance category: reclassification of cancer type (54.5%, n=18); reclassification to a non-malignant condition (33.3%, n=11); non-confirmation of suspected relapse (12.1%, n=4). Percentages represent proportions of discordant cases only, not of the overall cohort (n=169).

Overall diagnostic concordance was 80.5% (n=136), with discordance in 19.5% (n=33) (**Figure 1B**): reclassification of cancer type (54.5%, n=18), reclassification to a non-malignant condition (33.3%, n=11), and non-confirmation of suspected relapse (12.1%, n=4). (**Figure 1C**).

### Disease stage at presentation

Because leukemia is systemic by definition and standard SEER staging does not apply to retinoblastoma, stage at presentation was assessed separately by diagnostic category among solid tumors, lymphomas, and CNS tumors (n=101; non-malignant conditions, leukemia, and retinoblastoma excluded from this denominator). Distant disease was present in 44.4% (n=24) of solid tumors, 65.2% (n=15) of lymphomas, and 12.5% (n=3) of central nervous system (CNS) tumors; localized disease in 31.5% (n=17), 4.3% (n=1), and 87.5% (n=21), respectively. All leukemia patients (n=40) presented with systemic disease at diagnosis. Retinoblastoma (n=6) was staged using the IIRC rather than SEER: 1 Group C, 1 Group D, and 4 Group E (67% high-risk) (**Table 2**).

**Table 2:** Final diagnosis, SEER stage, and diagnostic concordance (N = 169).

Characteristic	N (%)
Diagnosis category at KHCC	
**Solid tumors**	54 (32.0%)
Bone sarcoma	11 (6.5**%**)
Neuroblastoma	13 (7.7%)
Rhabdomyosarcoma	6 (3.5%)
Mucoepidermoid carcinoma	1 (0.6%)
Germ cell tumor	7 (4.1%)
Inflammatory myofibroblastic tumor	1 (0.6%)
Renal tumors	6 (3.5%)
Pancreatic tumors	1 (0.6%)
Liver tumors	1 (0.6%)
Thyroid cancer	3 (1.8%)
Nasopharyngeal carcinoma	1 (0.6%)
Giant cell tumor	1 (0.6%)
Non-RMS soft tissue sarcoma	1 (0.6%)
**Acute leukemias**	40 (23.7%)
B-cell acute lymphoblastic leukemia	28 (16.6%)
T-cell acute lymphoblastic leukemia	6 (3.6%)
Acute myeloid leukemia	4 (2.4%)
Mixed phenotype acute leukemia	1 (0.6%)
Myeloid sarcoma	1 (0.6%)
**Lymphomas**	23 (13.6%)
Hodgkin lymphoma	18 (10.7%)
Non-Hodgkin lymphoma	5 (3.0%)
**CNS tumors**	24 (14.2%)
Low-grade glioma	9 (5.3%)
High-grade glioma	6 (3.6%)
Ependymoma	2 (1.2%)
Medulloblastoma	4 (2.4%)
CNS germ cell tumor	1 (0.6%)
Craniopharyngioma	2 (1.2%)
**Hematologic disorders**	4 (2.4%)
Aplastic anemia	2 (1.2%)
Transfusion-dependent thalassemia	2 (1.2%)
**Histiocytic disorders**	7 (4.1%)
Langerhans cell histiocytosis	4 (2.4%)
Hemophagocytic histiocytosis	3 (1.8%)
**Retinoblastoma**	6 (3.6%)
**Other/non-cancer conditions**	11 (6.5%)
Healthy	5 (3.0%)
Congenital heart disease	1 (0.6%)
Other eye disorders	3 (1.8%)
Other non-malignant disorders	2 (1.2%)
Stage at presentation¹ (solid tumors, lymphoma, CNS tumor only; n=101)	
Solid tumors (n=54)	
Localized	17 (31.5%)
Regional	9 (16.7%)
Distant	24 (44.4%)
Not applicable²	4 (7.4%)
Lymphoma (n=23)	
Localized	1 (4.3%)
Regional	6 (26.1%)
Distant	15 (65.2%)
Not applicable²	1 (4.3%)
CNS tumor (n=24)	
Localized	21 (87.5%)
Regional	0 (0%)
Distant	3 (12.5%)
**Acute leukemia (n=40)**	Not staged — systemic disease by definition
Retinoblastoma, IIRC Group³ (n=6)	
Group C	1 (16.7%)
Group D	1 (16.7%)
Group E (high-risk)	4 (66.7%)
Diagnosis concordance between referral and final KHCC diagnosis	
Concordant	136 (80.5%)
Discordant	33 (19.5%)

¹Stage classification (Localized/Regional/Distant) applied only to solid tumors, lymphomas, and CNS tumors; leukemia is not staged, as it is systemic by definition; retinoblastoma is staged separately using the International Intraocular Retinoblastoma Classification (IIRC) rather than SEER, as SEER’s localized/regional/distant framework does not capture intraocular disease extent. ²”Not applicable” (n=5 total) comprises: Pt 127 (bone sarcoma), lost to follow-up before staging or diagnostic confirmation could be completed; inflammatory myofibroblastic tumor and giant cell tumor, entities for which the SEER localized/regional/distant framework does not cleanly apply; and 2 patients in whom suspected relapse was ruled out, leaving no active tumor to stage. ³IIRC Group A–E reflects intraocular tumor extent (seeding, retinal detachment risk); Group D–E indicates high-risk disease. CNS, Central nervous system; IIRC, International Intraocular Retinoblastoma Classification; KHCC, King Hussein Cancer Center; RMS, Rhabdomyosarcoma; SEER, Surveillance, Epidemiology, and End Results.

### Critically ill subgroup (PICU patients)

PICU-admitted patients (13.6%, n=23) had numerically higher rates of active or progressive disease (57% vs 36%, p=0.07) and significantly higher rates of distant disease (69% vs 37.5%, p=0.038) and infection at presentation (43% vs 8%, p<0.001). Rates of diagnostic discordance were numerically higher in the PICU group (30% vs 18%), though this did not reach statistical significance (p=0.12). Only 17% of PICU patients had received prior therapy in Gaza versus 55% of non-PICU patients (p<0.001) (**Table 3**).

**Table 3:** Clinical Characteristics of PICU (N = 23) vs. non-PICU patients (N = 146).

Characteristic	PICU		Non-PICU		P-value^1^
	N	%	N	%	
Male sex	18	78%	87	60%	—
Median age (range), years	5.5 (0.04–17)		6.9 (0.01–21)		—
Prior cancer-directed therapy in Gaza	4	17%	81	55%	<0.001
Active/progressive disease	13	57%	53	36%	0.0709
Distant disease (solid tumors/lymphoma/CNS tumor only)	9	69%	33	37.5%	0.038
Diagnostic discordance	7	30%	26	18%	0.12
Underweight	6	26%	38	26%	1.00
Infection at presentation^2^	10	43%	12	8%	<0.001
Mechanical ventilation	2	8.7%	0	0%	—
Mortality	11	48%	13	9%	<0.001
Early mortality (<30 days)	4	17%^3^	0	0%	—
Median time to death (range), days	61 (3–454)		182 (50–456)		—

¹p-values calculated using Fisher’s exact test for categorical variables and the Mann–Whitney U test for continuous variables, ^2^Infection defined as documented bacterial, viral, or fungal infection at presentation. ^3^Represents 36% of deaths within the PICU subgroup. Distant disease is assessed only among solid tumors, lymphomas, and CNS tumors (SEER-eligible categories; PICU n=13, non-PICU n=88); leukemia, retinoblastoma, and non-malignant conditions are excluded from this denominator (see **Table 2**). Active/progressive disease reflects disease status at time of arrival to KHCC (columns AA/AB of source data), distinct from **Table 1**’s post-evaluation status. PICU, pediatric intensive care unit; SEER, Surveillance, Epidemiology, and End Results.

Mortality in the PICU subgroup was 48% (n=11), with early mortality (≤30 days) in 17% (n=4) — all four early deaths in the entire cohort. Median time to death was 61 days versus 182 days in non-PICU patients; the majority of deaths were attributable to progressive disease.

### Infectious complications

Infectious complications affected 13% (n=22) at presentation, concentrated among PICU patients (43%). Pathogens included multi-drug resistant (MDR) organisms (Methicillin-resistant Staphylococcus aureus (MRSA), Vancomycin-resistant Enterococcus (VRE), Extended-spectrum beta-lactamase-producing Escherichia coli (E.coli) and carbapenem-resistant E. coli, Klebsiella pneumoniae, Acinetobacter baumannii, Pseudomonas aeruginosa) and viral infections including Coronavirus Disease of 2019 (COVID-19) and rhinovirus/enterovirus. Varicella was confirmed in 6% (n=10), occurring in immunocompromised patients in the context of documented disruption of routine immunization programs in Gaza during the conflict period. Individual vaccination histories were not available in this cohort; a direct causal link between immunization disruption and this specific outbreak cannot be established from the data presented.

### Nutritional and psychosocial status

Nutritional deficiency was frequently observed among tested patients, though testing rates varied by marker: underweight status (weight-for-age <5th percentile) was assessed in 167/169 (98.8%) and present in 26.3% (n=44/167); vitamin D was measured in 149/169 (88.2%) and deficient (<20 ng/mL) in 50.3% (n=75/149); albumin was measured in 167/169 (98.8%) and low (<3.5 g/dL) in 9.6% (n=16/167). Pre-albumin was measured in only 39/169 patients (23.1%), reflecting selective ordering in patients with clinical concern for malnutrition; among those tested, 89.7% (n=35/39) had low values (<20 mg/dL), likely reflecting both this ascertainment bias and the acute-phase suppression of pre-albumin by active malignancy and inflammation, independent of chronic nutritional status. (**Figure 2**). Clinically significant psychological distress (DTS ≥6) was present in 41.4% (n=70).

**Figure 2:**
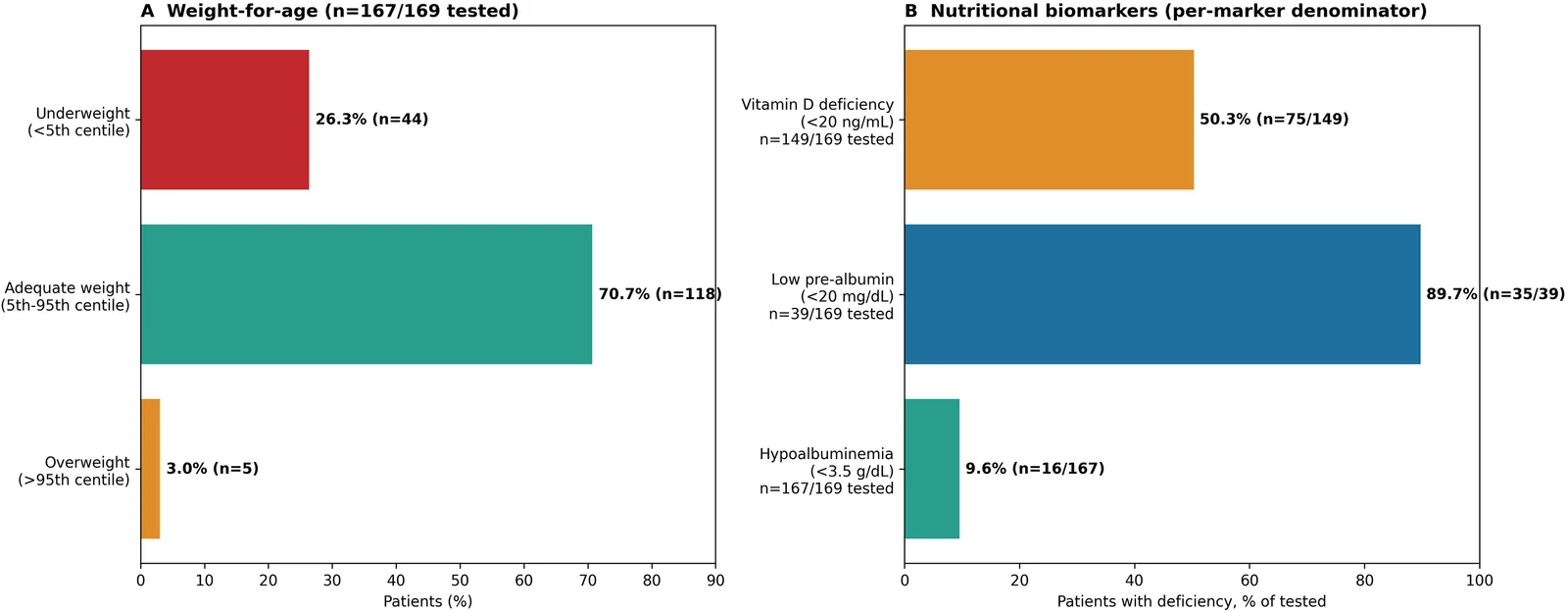
Nutritional status of displaced pediatric patients from Gaza at presentation to KHCC. **(A)** distribution of weight-for-age percentile categories (n=169), classified as underweight (<5th percentile), adequate weight (5th–95th percentile), and overweight (>95th percentile). **(B)** prevalence of laboratory-confirmed nutritional deficiencies. Deficiency thresholds: vitamin D <20 ng/mL; pre-albumin <20 mg/dL; albumin <3.5 g/dL; vitamin B12 <200 pg/mL. KHCC, King Hussein Cancer Center.

### Caregiver burden and system-level challenges

A total of 431 companions accompanied the 169 patients (median 2, range 1–10); (62%, n=268) were female. Siblings constituted the largest group (56%, n=239), followed by parents (40%, n=173), with other relatives accounting for 4% (n=19). The relationship structure of accompanying companions is reported descriptively; the study does not have data on primary caregiver identity or the specific reasons for companion composition. Administrative and logistical barriers — documentation requirements, movement restrictions, and evacuation delays — further complicated treatment initiation.

### Survival outcomes

During follow-up, (Median 8.0; IQR 5.2–22.0; months) data lock March 31, 2026), 24 deaths were recorded (14.2%); early mortality was 2.4% (n=4), all in the PICU subgroup. Most deaths (83%, n=20) were due to progressive disease; 17% (n=4) to infectious or treatment-related complications. Given the short median follow-up, outcomes are reported as 1-year and 2-year landmark rather than 5-year projections. The 1-year OS was 81.3% (95% CI: 72.1%–87.7%) and 2-year OS was 75.7% (95% CI: 64.6%–83.7%); 1-year EFS was 76.5% (95% CI: 67.0%–83.6%) and 2-year EFS was 70.6% (95% CI: 59.4%–79.3%). (**Figure 3A**). These estimates are consistent with the observed follow-up period; the observation period spanned approximately 28 months and estimates beyond this point should be interpreted with caution.

**Figure 3:**
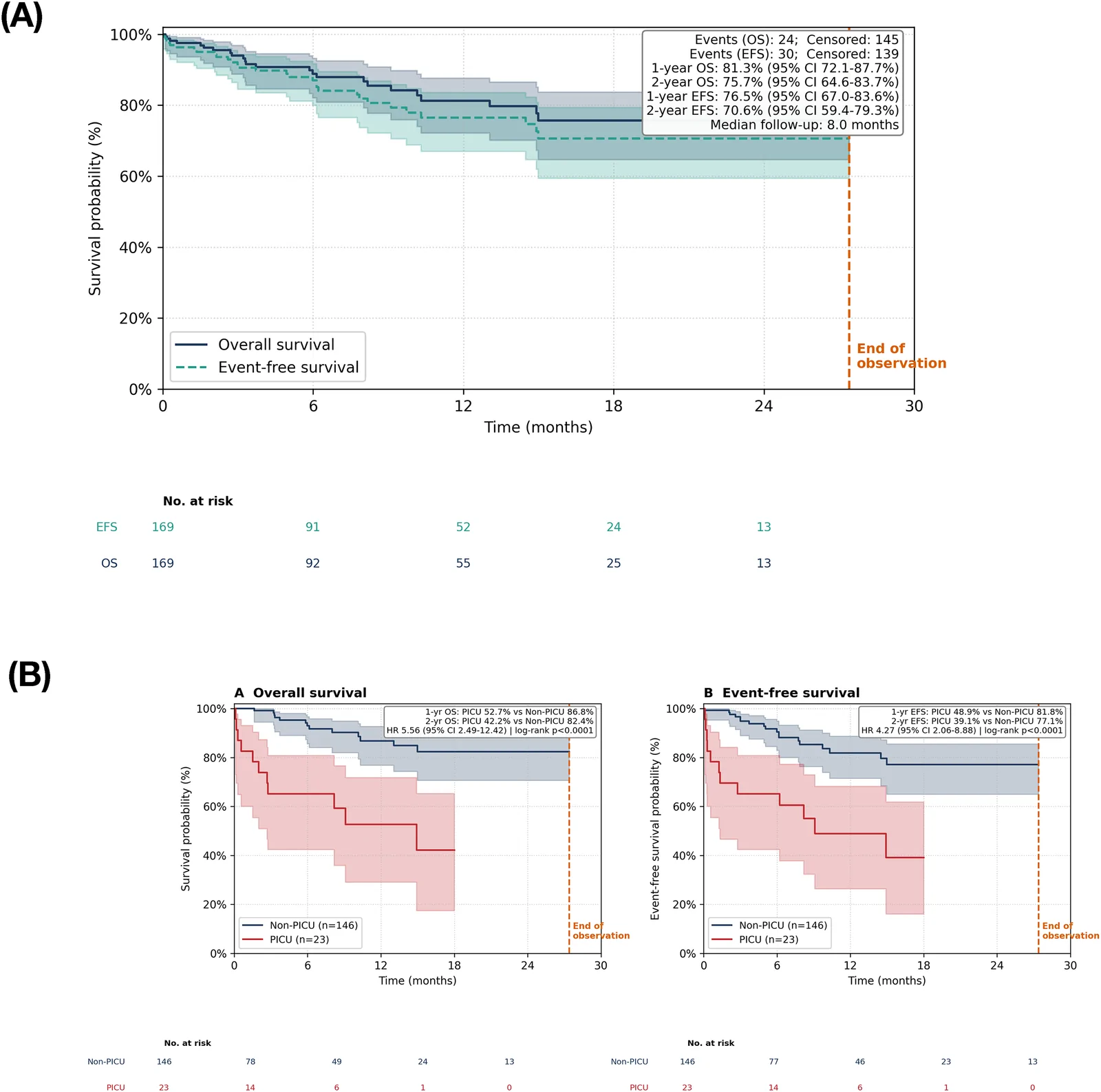
**(A)** Kaplan–Meier estimates of overall survival (OS) and event-free survival (EFS) for the overall cohort (n=169). Given the short median follow-up (8.0 months), outcomes are reported as 1-year and 2-year landmark estimates. The estimated 1-year OS was 81.3% (95% CI: 72.1%–87.7%) and 2-year OS was 75.7% (95% CI: 64.6%–83.7%); 1-year EFS was 76.5% (95% CI: 67.0%–83.6%) and 2-year EFS was 70.6% (95% CI: 59.4%–79.3%). Median OS and EFS were not reached. Shaded areas represent 95% confidence intervals (Greenwood method). The dotted vertical line indicates the end of the study observation period. KHCC, King Hussein Cancer Center; OS, overall survival; EFS, event-free survival; CI, confidence interval. **(B)** Kaplan–Meier survival curves stratified by clinical status at presentation (PICU vs non-PICU). Panel A: overall survival; Panel B: event-free survival. The estimated 1-year OS was 52.7% in the PICU group (n=23) versus 86.8% in the non-PICU group (n=146), and 2-year OS was 42.2% versus 82.4% (HR 5.56, 95% CI: 2.49–12.42; log-rank p<0.0001). The estimated 1-year EFS was 48.9% versus 81.8%, and 2-year EFS was 39.1% versus 77.1% (HR 4.27, 95% CI: 2.06–8.88; log-rank p<0.0001). Shaded areas represent 95% confidence intervals (Greenwood method). The dotted vertical line indicates the end of the study observation period. HR estimated by univariable Cox proportional hazards model. PICU, pediatric intensive care unit; HR, hazard ratio; CI, confidence interval; OS, overall survival; EFS, event-free survival.

Among PICU patients, 1-year OS was 52.7% versus 86.8% and 2-year OS was 42.2% versus 82.4% (HR = 5.56, 95% CI: 2.49–12.42, p<0.0001); 1-year EFS was 48.9% versus 81.8% and 2-year EFS was 39.1% versus 77.1% (HR = 4.27, 95% CI: 2.06–8.88, p<0.0001). (**Figure 3B**) This difference in survival outcomes between PICU and non-PICU patients reflects the substantially greater disease burden, higher rates of metastatic disease, and greater infectious morbidity present in PICU patients at presentation; the univariable Cox model is unadjusted for these and other confounders, and the observed association does not isolate delayed access to care as the sole contributing factor.

## Discussion

This study describes the clinical characteristics and early outcomes of children evacuated from Gaza during active conflict and treated at KHCC between November 2023 and March 2026, and is, to our knowledge, one of the largest cohort studies of conflict-evacuated children with cancer at a regional oncology center. The observed clinical profile — advanced disease at presentation, high rates of diagnostic discordance, nutritional deficiency, and psychosocial distress — is consistent with the documented effects of healthcare system disruption in conflict-affected settings (1, 3, 5, 6). These findings should be interpreted as descriptive observations in a conflict-evacuated cohort; the observational study design without a pre-conflict or non-conflict comparator group does not permit causal attribution of individual clinical findings to the conflict itself.

A central observation is the substantial interval between symptom onset and KHCC presentation, with a median of three months. This interval encompasses the full care pathway in Gaza, including prior cancer-directed therapy received by half of patients; it therefore reflects the total elapsed time to specialist evaluation at KHCC rather than a period of continuous diagnostic absence, though it was not always accurate. The observed distribution of advanced disease at presentation is consistent with patterns documented in conflict-affected settings in the published literature (5, 7, 8, 11, 12), though the relative contributions of the symptom-onset interval, prior treatment in Gaza, and underlying disease biology to stage at presentation cannot be disaggregated in this study. The temporal pattern of referrals over the 28-month study period is reported descriptively in **Figure 1**; formal analysis of temporal trends was not performed and claims about progressive deterioration of referral capacity are not made. The 19.5% rate of diagnostic discordance between referring institution and KHCC diagnoses is a clinically significant finding in a cross-border referral context. Limited access to immunohistochemistry, molecular diagnostics, and advanced imaging is associated with diagnostic discordance in resource-limited settings (20, 21). Among the 33 discordant cases, reclassification of cancer type occurred in 54.5% (n=18), reclassification to a non-malignant condition in 33.3% (n=11), and non-confirmation of suspected relapse in 12.1% (n=4). For cases with documented prior therapy in Gaza, the appropriateness of that treatment in relation to the final KHCC diagnosis cannot be determined from the available data. These findings support the case for diagnostic quality assurance — pathological review and multidisciplinary tumor board evaluation — as a component of cross-border oncology referral programs (21–23).

Patients requiring PICU admission at presentation (13.6%) had markedly worse survival outcomes than non-PICU patients: 1-year OS 52.7% versus 86.8% and 1-year EFS 48.9% versus 81.8% (HR 5.56 and 4.27, respectively; P<0.0001). This survival difference reflects the substantially greater disease burden, higher rates of metastatic disease, and greater infectious morbidity in PICU patients at presentation. As the Cox model is univariable and unadjusted for these confounders, the association between PICU admission and worse survival outcomes is consistent with confounding by disease severity and indication; it does not demonstrate that delayed access to care was the primary driver of mortality in this group (3, 7).

Malnutrition — affecting 26.3% by weight-for-age and 50.3% by vitamin D levels among those assessed — is associated with reduced chemotherapy tolerance, increased infectious susceptibility, and worse survival in pediatric oncology (24, 25). These nutritional findings reflect a combination of underlying malignancy, prior treatment, and displacement-related factors that cannot be disaggregated in this study. Psychological distress was present in 41.4% of assessed patients. Contributing factors may include illness burden, displacement, family separation, and conflict exposure, although their relative contributions could not be disentangled in this study (14, 15). Nutritional assessment and psychosocial support must function as core clinical infrastructure, not supplementary services, at any center receiving conflict-displaced children with cancer. Infectious complications — including MDR organisms — were disproportionately concentrated among the most critically ill (26–28). Varicella was confirmed in 10 patients, including immunocompromised individuals, in the context of documented disruption of routine immunization programs in Gaza during the conflict period (27, 29). Individual vaccination histories were not available in this cohort; a direct causal relationship between immunization disruption and the observed varicella cases cannot be established. Nevertheless, these findings support systematic vaccination screening and urgent catch-up immunization at the earliest clinically safe opportunity upon arrival at receiving centers (9).

This study identifies caregiver instability and system-level administrative barriers as clinically relevant challenges in humanitarian oncology (25, 30, 31). Siblings constituted the largest group of accompanying companions (56%); companion data are reported descriptively and do not permit conclusions about primary caregiver capacity Administrative barriers — security clearances, documentation mandates, and movement restrictions — impose evacuation delays that have clinical implications (10). Structured repatriation planning, including emergency re-evacuation protocols and remote follow-up, should be standard in humanitarian oncology programs (10).

The 1-year OS of 81.3% and 2-year OS of 75.7% observed in this cohort are encouraging and compare favorably with data from equivalent humanitarian settings (12), demonstrating that meaningful survival is achievable even with late, malnourished, advanced-disease presentations — provided patients reach a capable receiving center. It is important to note that these outcomes describe only children who successfully navigated evacuation: those who died before evacuation, for whom evacuation was impossible, or in whom cancer was never diagnosed represent an unquantifiable additional burden not captured in this study. The observed survival reflects multiple contributing factors including the underlying disease distribution, prior treatment received in Gaza, the selection effect of the evacuation process, and the care delivered at KHCC; the independent contribution of each factor cannot be isolated in this observational study.

The KHCC–SJCRH collaboration illustrates the role of international partnerships in enabling continuity of pediatric oncology care for conflict-displaced children (13). In this cohort, 59% of patients were supported through King Hussein Cancer Foundation (KHCF) resources and 41% through SJCRH support. Although the sustainability of these funding arrangements was not formally evaluated in this study, the reliance on philanthropic and institutional support highlights the importance of predictable, long-term financing mechanisms for pediatric cancer care in humanitarian settings.

The following recommendations are informed by the findings of this cohort together with the broader humanitarian oncology literature; several of these areas, including evacuation logistics, telemedicine infrastructure, and financing mechanisms, were not directly evaluated in this study. Based on these findings and the broader literature, four priorities warrant consideration: First, protected, legally recognized medical evacuation corridors for children with cancer are required under International Humanitarian Law (10) — evacuation delays have direct clinical consequences for critically ill patients. Second, systematic diagnostic quality assurance must be integrated into all cross-border referral programs, including standardized pathological review, tumor board confirmation, and telemedicine-based support (21–23).Third, pediatric oncology must be explicitly named and resourced within global humanitarian emergency health frameworks, including the WHO Global Initiative for Childhood Cancer (6), as an acute life-threatening emergency — not a chronic post-conflict issue. Fourth, philanthropic funding dependence must be replaced by predictable multilateral mechanisms through WHO, UNRWA, or equivalent structures to ensure continuity independent of donor cycles (6, 13).

Without urgent global action, conflict-affected settings risk reversing decades of progress in childhood cancer survival. For every child in this cohort, an unknown number did not reach care. Ensuring equitable access to timely diagnosis and treatment for children with cancer in conflict settings is a central imperative for global oncology — and one this evidence supports.

## Limitations

The most fundamental limitation is an unquantifiable referral and survival bias: this cohort includes only patients who successfully navigated evacuation from Gaza to Jordan. Children who died before evacuation was possible, those for whom physical access, administrative clearance, or family capacity made evacuation unachievable, and those in whom cancer was never suspected or diagnosed represent a wholly invisible denominator. The findings therefore characterize outcomes in a selected subpopulation and cannot be generalized to the full scope of pediatric oncologic burden in Gaza. This selection bias is inherent to any conflict-evacuated cohort and should be considered when interpreting all reported outcomes, particularly survival estimates.

The retrospective design and reliance on electronic medical records introduce information bias regarding pre-referral treatment details, prior diagnostic workup, and symptom onset documentation. No pre-conflict cohort and no contemporaneous non-conflict comparison group are available, precluding causal inference regarding the specific contribution of the conflict to the observed clinical findings. The reported 1-year and 2-year OS and EFS estimates are derived from the observed follow-up period; confidence intervals are correspondingly wide, particularly in the PICU subgroup, and should be interpreted with caution pending follow-up maturation. No multivariable Cox regression was performed due to sample size constraints and diagnostic heterogeneity; the reported hazard ratio for PICU admission is univariable and unadjusted for disease stage, tumor type, and other clinical confounders. Caregiver and administrative burden data were collected through routine clinical documentation rather than systematic prospective assessment, likely underestimating their true prevalence. EFS should be interpreted in the context of the study-specific time origin and heterogeneous baseline disease status; patients presenting with relapse or progression were not counted as having an EFS event at baseline, and therefore these estimates are not directly comparable with conventional diagnosis-to-event survival estimates. As a single-center experience at a well-resourced referral center supported by an international partnership, generalizability to humanitarian oncology programs with more limited resources may be constrained.

## Conclusion

Children evacuated from Gaza during active conflict presented to KHCC with a high burden of advanced or active disease, diagnostic discordance, nutritional deficiency, and psychosocial distress — findings consistent with the documented effects of healthcare system disruption in conflict-affected settings, though the observational design without a comparator group precludes definitive causal attribution. The marked difference in survival between PICU-admitted and non-PICU patients reflects the concentration of mortality risk in the most clinically burdened subgroup, with confounding by disease severity acknowledged. At the same time, this study demonstrates that access to comprehensive, multidisciplinary care at a regional center of excellence — sustained by committed international partnerships — is associated with meaningful and encouraging survival outcomes even in the context of profound clinical vulnerability. These outcomes, however, describe only those children who successfully navigated evacuation: for every patient in this cohort, an unknown number did not reach care. The true oncologic burden of this conflict on children remains incompletely characterized and is almost certainly substantially greater than our data reflect.

Urgent, coordinated global action is required: to establish protected medical evacuation corridors; to integrate pediatric oncology into humanitarian emergency response frameworks; to invest sustainably and predictably in regional centers of excellence (13); to strengthen diagnostic and treatment capacity in conflict-affected settings (9, 10). Children with cancer in conflict-affected settings cannot wait for peace to receive equitable access to life-saving care. Ensuring that they receive it — now, in Gaza, and in every future conflict — is not a charitable aspiration: it is an obligation of global solidarity, and one this evidence demands we meet.

## Data Availability

The original contributions presented in the study are included in the article/**Supplementary Material**. Further inquiries can be directed to the corresponding authors.
